# Novel adenoviruses detected in British mustelids, including a unique *Aviadenovirus* in the tissues of pine martens (*Martes martes*)

**DOI:** 10.1099/jmm.0.000546

**Published:** 2017-07-28

**Authors:** David Walker, William F. Gregory, Dylan Turnbull, Mara Rocchi, Anna L. Meredith, Adrian W. Philbey, Colin P. Sharp

**Affiliations:** ^1^​ Royal (Dick) School of Veterinary Studies and The Roslin Institute, University of Edinburgh, Easter Bush, Edinburgh EH25 9RG, UK; ^2^​ Moredun Research Institute, Pentlands Science Park, Bush Loan, Edinburgh EH26 0PZ, UK

**Keywords:** adenovirus, pine marten, otter, cross-species transmission, high-throughput sequencing

## Abstract

Several adenoviruses are known to cause severe disease in veterinary species. Recent evidence suggests that canine adenovirus type 1 (CAV-1) persists in the tissues of healthy red foxes (*Vulpes vulpes*), which may be a source of infection for susceptible species. It was hypothesized that mustelids native to the UK, including pine martens (*Martes martes*) and Eurasian otters (*Lutra lutra*), may also be persistently infected with adenoviruses. Based on high-throughput sequencing and additional Sanger sequencing, a novel *Aviadenovirus*, tentatively named marten adenovirus type 1 (MAdV-1), was detected in pine marten tissues. The detection of an *Aviadenovirus* in mammalian tissue has not been reported previously. Two mastadenoviruses, tentatively designated marten adenovirus type 2 (MAdV-2) and lutrine adenovirus type 1 (LAdV-1), were also detected in tissues of pine martens and Eurasian otters, respectively. Apparently healthy free-ranging animals may be infected with uncharacterized adenoviruses with possible implications for translocation of wildlife.

Adenoviruses have been shown to infect a wide range of species. The vast majority of the literature relating to adenoviruses in mammals concerns those which infect human beings and non-human primates. Despite the high prevalence of infection with adenoviruses it is recognized that, particularly in human beings, adenoviruses uncommonly cause severe systemic disease and usually only in infants and the immunosuppressed [1, 2].

In veterinary medicine, some adenoviruses have been well characterized, primarily those which cause overt disease, including canine adenovirus types 1 (CAV-1 [3, 4]) and 2 (CAV-2 [5]), bovine adenoviruses [6, 7] and avian adenoviruses [8–10]. As has been shown in human beings and non-human primates [11, 12], adenoviruses in infected non-primate species can be detected in a wide range of tissues, in the presence of neutralizing antibodies and in the absence of gross pathology, which is suggestive of persistent infections [13, 14]. Some animals may also shed infectious virus for a prolonged period of time after infection [14, 15]. However, the mechanisms which allow adenoviruses to persist in the tissues of hosts have not been fully elucidated.

In non-domestic species, the prevalence of adenoviruses and the characterization of disease that they may cause has not been extensively explored, particularly in free-ranging species with no perceived commercial benefit. Intermittent studies have demonstrated the presence of adenoviruses in wildlife in the UK. For example, adenoviruses have been detected in both red squirrels (*Sciurus vulgaris*) and grey squirrels (*S. carolinensis*), leading to the recommendation that adenoviruses should be considered in squirrel conservation programmes [16]. Furthermore, it has been reported that red foxes play a major role as a wildlife reservoir of CAV-1 in the UK [14] and Italy [17] and may be a disease risk for dogs.

There are several species of mustelid in the UK, including Eurasian otters (*Lutra lutra;* hereafter referred to as otters) and pine martens (*Martes martes*). In the UK, otters are widespread and frequent rivers and coastal areas, where they prey mainly on fish and crustaceans, but also small reptiles, birds and other mammals. Pine martens are a more elusive species and are uncommon in the UK due to historical persecution, despite being widespread across mainland Europe. Pine martens feed on a wide range of available prey species including birds, small mammals, reptiles and invertebrates, in addition to nuts and berries. It is important to establish whether adenoviruses, along with other novel pathogens, have an impact on these species and whether they should be considered in conservation policies. For example, a recent translocation project involving pine martens was initiated in the UK in an attempt to increase the total population [18]. The home ranges of different species of mustelids, as well as other predators such as red foxes (*Vulpes vulpes*), may overlap and there is potential for cross-infection with pathogens via indirect contact with urine, faeces and infected fomites [19], particularly if translocated pine martens are migrating during the establishment of new colonies.

Moreover, it is also important to establish whether mustelids are reservoirs of disease, including uncharacterized adenoviruses, for other wildlife and for domestic mammals, including ferrets (*Mustela putorius furo*) and dogs. Severe disease has been identified in a single captive otter in Seoul Grand Park Zoo, South Korea; this was thought to have been caused by CAV-1, although sequencing was based on a relatively short amplicon [20]. Disease caused by skunk adenovirus type 1 (SkAdV-1) has been reported in a single striped skunk (*Mephitis mephitis*; family *Mephitidae*) in Canada [21]. However, to the authors’ knowledge, no adenoviruses have been detected previously in free-ranging species in the family *Mustelidae* in the UK. In southwest France, some mustelid species had antibodies against adenovirus, but these were not necessarily specific towards the test antigen (CAV-1) [22]. Due to serological cross-reactivity amongst adenoviruses [23, 24], there is a possibility that novel adenoviruses may be present in free-ranging mustelids. In the present study, we have screened tissues from pine martens and otters for the presence of adenoviruses.

Nine otter carcasses were obtained from the Shetland Islands, Scotland, UK, and a total of 14 pine marten carcasses were obtained from mainland Scotland, mainly in the Aberdeen region. The animals had died as a result of road traffic accidents or were found dead due to inapparent causes. During the post-mortem examinations of both species, a range of tissues were collected (Table S1, available in the online Supplementary Material). There were no signs of gross pathological changes that might indicate the presence of viral disease. The tissues were unsuitable for histological examination because the carcasses had been frozen and thawed, and were subject to varying degrees of autolysis. DNA was extracted from samples using the DNeasy Blood and Tissue Kit (Qiagen, Hilden, Germany) and the E.Z.N.A Stool DNA Kit (Omega Bio-tek, Norcross, Georgia, USA). Procedures to minimize cross-contamination were followed, and DNA from faecal samples and tissues was extracted separately.

The livers from the otters and pine martens were initially screened using an adenovirus consensus nested PCR, which amplifies adenovirus DNA polymerase. This was adapted from the original method of Wellehan *et al.* [25], previously described by Walker *et al*. [4]. Following gel electrophoresis of the second-round products, amplicons from positive samples were sequenced directly (Edinburgh Genomics, University of Edinburgh, UK). Sequences were analysed using the Basic Local Alignment Search Tool (blast) [26].

Using the nested PCR, two different novel adenovirus DNA polymerase sequences were detected in two pine marten livers, tentatively named marten adenovirus type 1 (MAdV-1) and marten adenovirus type 2 (MAdV-2). Pooled DNA from three adenovirus-positive otter livers revealed another novel adenovirus sequence, tentatively named lutrine adenovirus type 1 (LAdV-1; the detected sequences not submitted to GenBank are available in the Supplementary Material).

Unexpectedly, the MAdV-1 DNA polymerase nucleotide sequence shared most identity (71 %) with an *Aviadenovirus*, fowl adenovirus type 3 (GenBank KT862807.1). Kidney and faeces from the same pine marten, and the faeces of an additional pine marten, were positive for the same adenoviral sequence using the consensus adenovirus PCR. MAdV-2, detected in the liver of one pine marten, shared 75 % identity with vespertilionid adenovirus type 1 (GenBank KM043089.1), a *Mastadenovirus*, but was not present in any other samples or animals. The nucleotide sequence from LAdV-1 shared 81 % identity with Indian flying fox adenovirus type 5 (GenBank KC692421.1), a *Mastadenovirus*.

Additional novel sequences from the faecal samples of five pine martens, included in blast analyses, shared most identity with adenoviruses originating from bird, lizard and mammal hosts. These were not present in the tissues (liver, kidney or lung) of the same pine martens. It was assumed that this DNA was present as a result of digestion of adenovirus-infected prey by the pine martens shortly before death, and could have represented part of a mixed population of several adenoviruses in the faeces. These sequences were not analysed further in this study.

Attempts to isolate and purify adenoviruses from pine marten and otter tissues by cell culture inoculation were unsuccessful. Extractions containing the adenoviral DNA present in multiple individuals (i.e. MAdV-1 and LAdV-1) were prepared directly from the liver of pine marten 10 and the kidney of otter 08 for high-throughput sequencing (HTS; Table S1), performed at the Centre for Genomic Research (CGR), University of Liverpool, UK. The DNA library was prepared using the Tru-seq Nextera system (Illumina, San Diego, California, USA) and 250 base pair (bp) paired-end reads were obtained using the MiSeq platform (Illumina). The initial processing of data, read mapping and assembly were performed by CGR. Remaining non-assembled reads were screened and assembled using Geneious 9.1.6 software (Biomatters, Auckland, New Zealand). Contigs were then analysed using blast to identify adenovirus sequences from the two mustelid samples. Adenovirus hits were then imported into mega6 [27] and SSE [28] software for alignment to selected reference genomes. 18 contigs generated from the MiSeq reads were identified to be part of the MAdV-1 genome (including sequences encoding putative IVa2, pTP, 52K, pIIIa, penton, pVI, DNA-binding protein and pVIII). All of these contigs from the MAdV-1 genome had the closest identity to aviadenoviruses using blast.

From the otter sample, only three unpaired 250 bp reads showed significant similarity to *Mastadenovirus* sequences by blast analysis. These reads were assumed to be from LAdV-1 and showed 72–81 % identity to the hexon, DNA polymerase and 52K coding regions of bat adenovirus WIV13 isolate (sequences are available in the Supplementary Material).

Additional PCR and Sanger sequencing (Edinburgh Genomics) was performed, using sequences from the putative MAdV-1 genome, in order to obtain sequences between contigs. The primers and PCR protocols used to obtain these sequences are described in the Supplementary Material. The complete predicted MAdV-1 hexon and DNA polymerase gene sequences were subsequently determined (GenBank KY705357–KY705358). Other assembled sequences from MAdV-1 were submitted to GenBank under accession numbers KY705359–KY705373.

Primers were designed from the MAdV-1 DNA polymerase gene to estimate the frequency of this virus amongst all samples obtained from the 14 pine martens included in the study using a specific nested PCR (Supplementary Material). The frequency of LAdV-1 amongst all samples obtained from the nine otters was estimated using the consensus adenovirus DNA polymerase nested PCR [4, 25]. Amplicons from positive samples were confirmed by Sanger sequencing (Edinburgh Genomics).

MAdV-1 sequences were detected in four of 12 (33.3 %) faecal samples from pine martens. Livers from two of 14 (14.3 %) pine martens were positive for MAdV-1; samples of kidney from the same animals were also positive (two of 13; 15.4 %), along with faecal samples. Overall, MAdV-1 was present in samples from four of 14 (28.6 %) pine martens. The PCR-positive animals included the initial positive pine marten subjected to HTS. LAdV-1 was detected in six of nine (66.6 %) livers and five of nine (55.6 %) kidneys from otters. Three otters were positive for LAdV-1 sequences in both the kidneys and liver. Overall, the frequency of LAdV-1 in otters was eight of nine (88.9 %) (Table S1; GenBank KY753135–KY753142).

On the basis of phylogenetic analysis, the MAdV-1 hexon and DNA polymerase genes group within the *Aviadenovirus* clade and appear to be most closely related to an ancestor of the contemporary fowl adenovirus group (Fig. 1). Although it is a limitation of the study that the full genome was not obtained by HTS, it is highlighted that the other contigs obtained by HTS, corresponding to other genome regions, also had the closest identity to aviadenoviruses using blast (GenBank KY705359–KY705373). This is a unique and unusual finding, because all aviadenoviruses discovered to date have been detected only in avian host species. The partial predicted amino acid sequences from LAdV-1 (and MAdV-2; GenBank KY753134) DNA polymerase shared most identity with the mastadenoviruses by blast analysis and upon phylogenetic reconstruction (not shown due to the limited sequence data obtained). This was predicted, since all known mastadenoviruses originate from mammalian hosts.

**Fig. 1.**
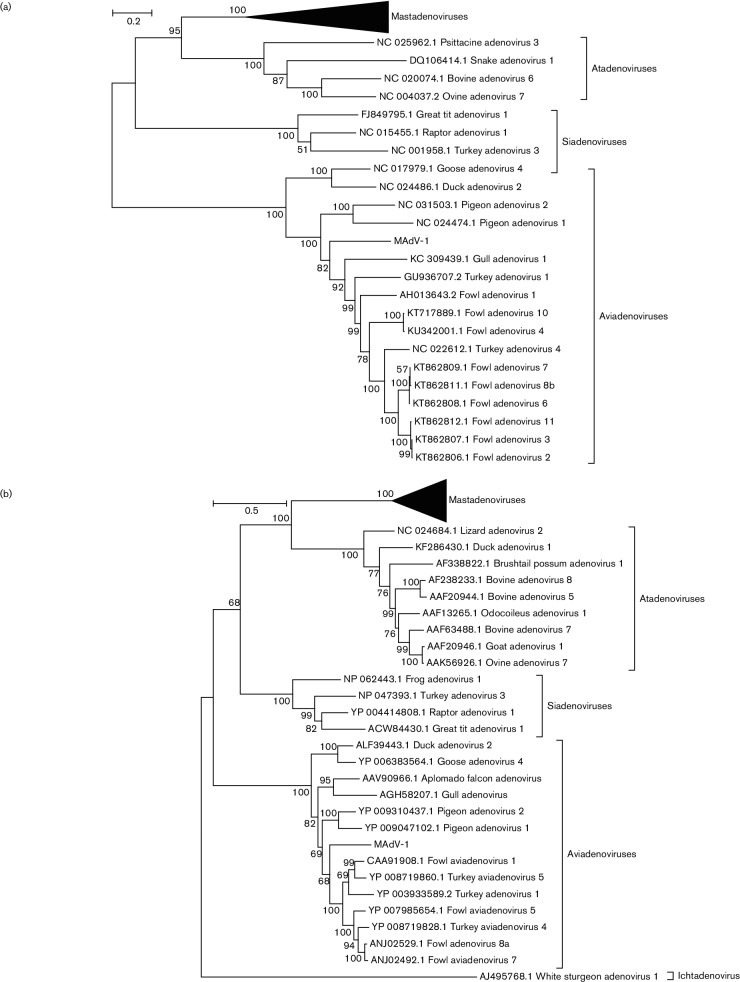
The predicted amino acid sequences of MAdV-1 DNA polymerase (a) and hexon (b) genes were imported into mega6. Sequences were aligned to predicted amino acid sequences of other adenovirus hexon and DNA polymerase genes using clustalw [37]. For each gene, the best maximum likelihood model was selected using mega6, which was then used to construct a phylogeny from 500 bootstrap replications.

The detection and sequencing of MAdV-1 in pine martens raises the possibility that a cross-species transmission event of an adenovirus between divergent predator and prey species may have occurred. It has been previously suggested that atadenoviruses may represent potential host-switching events, on account of the divergent host species in this branch [25]. Cross-species transmission events have also been suggested for closely related or associated species, such as simians and human beings [29]. An outbreak of fulminant pneumonia in human beings in contact with a New World monkey colony was the result of cross-species transmission of titi monkey adenovirus (TMAdV) [30]. Additionally, a captive otter was determined to have died of ‘infectious canine hepatitis’ caused by CAV-1 in Seoul Grand Park Zoo, although sequencing was based on a relatively short amplicon [20]. These cases may represent opportunistic cross-species transmission events.

It is possible that MAdV-1 could represent a historical host-switching event of an ancestor of the fowl adenoviruses, which has successfully established in a predator host. The *Aviadenovirus* may have become established in pine martens following predation of infected birds. The virus could have adapted to transmit among the new host species and may be endemic in Scottish pine martens. Another possibility is that MAdV-1 DNA sequences can be detected in the tissues of pine martens following predation of infected birds without true replicative infection taking place in the predator species. This could result from haematogenous dissemination of viral particles or DNA from the intestinal tract following ingestion of prey tissues, resulting in positive PCR results in pine marten tissues.

Several studies that aimed to estimate the prevalence of adenoviruses in wildlife only screen faecal samples because of their ready availability (e.g. from wild rodents [31, 32]). However, detection of adenoviruses in faeces alone (e.g. without evidence of disease) should be viewed with caution since the presence of adenoviral DNA may be due to ingestion of infected prey, with subsequent excretion of intact adenoviral capsids and/or DNA following gastrointestinal passage. In the current study, we detected an *Aviadenovirus* (MAdV-1) in both tissues and faeces in multiple animals and suggest that this most likely indicates the presence of infection in pine martens. Furthermore, we would have expected to detect multiple different aviadenoviruses of prey origin if pine martens were not the true host of MAdV-1. However, there is a need to screen tissue samples from prey species for the presence of MAdV-1 sequences.

It remains to be determined if and how MAdV-1 can spread among pine martens, although the faecal-oral route is likely, given the presence of the virus in faecal samples. Whether the virus can re-infect avian species cannot be determined until the virus is isolated. Furthermore, it is uncertain whether any disease is caused by MAdV-1. MAdV-2 and LAdV-1 closely resemble mastadenoviruses and may have evolved from other mastadenoviruses following co-speciation with their hosts [33].

The lack of gross lesions suggestive of viral infection in pine martens or otters implies that the animals were likely to be persistently infected. There is evidence for persistent infection with CAV-1 in red foxes, which are a wildlife reservoir of the virus for dogs [14]. Some other adenoviruses also have broad host ranges [20, 30, 34], so the possibility of transmission of a novel adenovirus from wild mustelids to domestic mustelids, such as ferrets, should be considered.

In conclusion, we have detected at least three novel adenoviral sequences in tissues and faeces from two species of mustelids in the UK, namely pine martens and otters. Further work is required to determine whether these are true mustelid adenoviruses that are able to undergo replication and transmission in their respective host species. Further efforts to isolate the viruses in cell culture are warranted. It is also important to establish the mechanisms that allow adenoviruses to establish persistent infections. The presence of persistent infection with potentially pathogenic viruses should be taken into consideration when translocating free-ranging animals between habitats [35, 36], and also when transferring captive animals amongst zoological collections.

## Supplementary Data

445 Supplementary File 1
