# Supplementary material for: Novel adenoviruses detected in British mustelids, including a unique Aviadenovirus in the tissues of pine martens (Martes martes)
Source: J Med Microbiol. 2017 Jul 28;66(8):1177–82. doi: 10.1099/jmm.0.000546 (PMC5817191; doi:10.1099/jmm.0.000546)
Supplement: Supplementary File 1 [file jmm-66-1177-s001.pdf]

Supplementary Materials

Novel adenoviruses detected in British mustelids, including a unique *Aviadenovirus* in the tissues of pine martens (*Martes martes*)

David Walker [a*], William F. Gregory [a], Dylan Turnbull [b], Mara Rocchi [b], Anna L. Meredith [a], Adrian W. Philbey [a], Colin P. Sharp [a].

[a] *Royal (Dick) School of Veterinary Studies and The Roslin Institute, University of Edinburgh, Easter Bush, Edinburgh EH25 9RG, UK*
[b] *Moredun Research Institute, Pentlands Science Park, Bush Loan, Edinburgh EH26 0PZ, UK*

* Corresponding author.
E-mail address: david.walker@roslin.ed.ac.uk

Preparation of tissues for high throughput sequencing (HTS)

DNA was prepared directly from tissue samples for HTS. 288 mg of liver from pine marten 10 and 500 mg of kidney from otter 08 were macerated using scalpel blades. Samples were transferred to microcentrifuge tubes with 500 μL of phosphate buffered saline (PBS) and glass or ceramic beads, then further disrupted using a vortex with horizontal adaptor (Mo Bio, Carlsbad, California, USA). The samples were then centrifuged at 12,100 x g for 5 min. The supernatant was extracted and filtered through a Millex-HA 0.45 μm syringe filter unit (Merck Millipore, Carrigtwohill, Co. Cork, Ireland). Residual supernatant in the filter membrane was displaced by syringing of an additional 500 μL PBS into the filter.

To increase the relative amount of encapsidated viral DNA to host DNA in the sample the following was added to each 600 μL of supernatant: 15 μL TURBO DNA-free DNase treatment (Life Technologies, Carlsbad, California, USA), 60 μL of 10x TURBO DNase buffer and 15 μL RNase A (Thermo Fisher Scientific, Waltham, Massachusetts, USA); this mixture was incubated for 90 min at 37 ºC. An equal volume of 'Buffer AL' from the DNeasy Blood and Tissue Kit (Qiagen, Hilden, Germany) was then added, followed by an equal volume of 100 % ethanol. The total volume was centrifuged through DNeasy spin columns (Qiagen). The extraction then followed the manufacturer's protocol for the DNeasy Blood and Tissue Kit (Qiagen). The consensus nested polymerase chain reaction (PCR) for adenovirus DNA polymerase [4, 25] was used to verify the presence of specific amplicons. DNA concentrations were determined using the Qubit 3.0 flourometric quantitation system (Thermo Fisher Scientific)

Novel adenovirus sequences not submitted to GenBank

The following sequences were detected in tissues, which were originally screened using the adenovirus consensus nested PCR [4, 25] but were not submitted to GenBank (due to their short length and/or latter sequencing from non-pooled DNA). The sequences are displayed in FASTA format, including a descriptor for 'isolate' source and tissue. The 'isolate' refers to the amplicon detected in a specific individual animal (PM = pine marten, O = otter; Supplementary Table S1).

>Marten adenovirus type 1 DNA polymerase partial CDS, isolate PM10 liver

CAATGTCAATGATGGTAATGACTTCATCATACAATGTCTCGTTTGTCCACACTAA

CCTCCCACCACGCCTGCTGCAAATGGGAGGTAGTGGATCGAGGTTTACCATGGAT

GGTGGGTAAGCATCCACTTTGAGTATAGCAGGCTTGATTCTAGAGTCAAAATAGC

TGATGTCTGGATGGTCCTGAAGAAGAACGTTGAGCTCTTCCACGTATTGTGCAGT

GTTGGCAGGATCTAGTGGCATACCGTGAGGCATCGGATGGGTAAGTGCTGAA

>Lutrine adenovirus type 1 DNA polymerase partial CDS, isolate pooled O7, O8, O9 livers

GCGGGATATACATGGCATCCCTCGTAATTTTCATTAGTCCAACAAAGTCTACCTC

CTTTTCTAGAACAAAAGGGAGGAAGGGGATCGAGATAAATTTCTTCTGGAGAAT

CCGCATCAATTGTAAATATTCCAGGTTTGAGATTTGGATCGAAATAATTAATCTT

TGATACCGTGCTTAACTTTTTTTCCCATTCTCTGATGGCCAGAGTTCGGTCGTATG

GGTTTAACGCTAAGCCTGAAGGTAGAGGATGGGTTAAAGCGCTG

The following sequences, designated as part of the lutrine adenovirus type 1 (LAdV-1) genome, are single 250 base pair (bp) reads obtained from HTS of kidney from otter 08. These were not submitted to GenBank because the 'pair' of the read did not adequately

sequence, were not part of assembled contigs and the small number of reads was determined to be unsuitable for submission to the Sequence Read Archive (SRA).

> Lutrine adenovirus type 1 DNA polymerase partial cds isolate O8 kidney

TGGCAATACTGCGCATTGTTTGATTTTTTTCAAAATCTGCTTTTTCTTTAGCTTGA
ATGTTTAACTCAACATACTCTTTAGCTATACATTTCCATTCTGAAAACACCGTACA
TCTTTCATCGGGAAGTAGTCTAACTTTCCACCCCCTATTGTGAAGTGTAATAACG
TCAATGGAAGTGGCAATTTCACCTCGTAAATTTTCATTAGTCCAACAAAGTCTAC
CTCCTTTTCTAGAACAAAATGGAGGAAG

> Lutrine adenovirus type 1 52K partial cds, isolate O8 kidney

AGGAAGTTGTAATTGGATTAATTTATTTGTGGGATTTTGTAGATGCTTATATTCAT
AATCCCTCGAGCAAAGTTTTAACCGCTCAACTTTTTTTAATTATTCAACACTCGAG
AGATGAAGGAATTTTTAAAGAATCCTTACTTAACATTACTTCAGAAGAAAATAAG
TGGCTAGTTGATTTGATAAACATTTTACAAACTATCGTAGTACAAGAAAGGACTT
TAAAAATTTCTGAAAAAGTTTCAGCTAT

> Lutrine adenovirus type 1 hexon partial cds, isolate O8 kidney

GCAATCTTGTAGATAGCTACATAAATATTGGCGCCCGTTGGTCTTTAGATGTAAT
GGATAATGTTAATCCTTTTAATCATCATAGAAATCCTGGATTAAGATATAGATCT
CAGCTATTAGGCAATGGTCGTTACTGTGAATTTCATATTCAGGTGCCTCAAAAAT
TTTTTGCAATCAAAAATCTCTTATTACTTCCTGGAACCTATAGTTACGAATGGTCC
TTTAGAAAAGACGTTAATATGATTTTACA

Screening of pine marten tissues for marten adenovirus type 1 (MAdV-1) using a specific nested PCR

A specific nested PCR was designed to screen pine marten tissues for MAdV-1. Primers were designed from the MAdV-1 DNA polymerase gene sequence. The forward and reverse first round primers were PMPolFouter (5'-GCTGATGTCTGGATGGTCCT-3') and PMPolRouter (5'-ATACCGAGCAATACCCATGC-3'), respectively. The second round primers were PMPolFinner (5'-TGCAGTGTTGGCAGGATCTA-3') and PMPolRinner (5'-CCGCACAGAGAGATGTTCAA-3'). DNA (5 µL from the first DNA extraction or 5 µL first round PCR product in the second round) was added to 31.8 µL H_2O, 0.2 µL (1 U) GoTaq G2 DNA polymerase (Promega, Madison, Wisconsin, USA), 10 µL 5x Green GoTaq reaction buffer (Promega), 1 µL deoxynucleotide triphosphates (dNTPs; final concentration 200 µM each dNTP), 1 µL first/second round forward primer (final concentration 200 nM) and 1 µL first/second round reverse primer (final concentration 200 nM). For both rounds of amplification, the PCR reaction conditions were 95 ºC for 1 min, followed by 45 cycles of 95 ºC for 1 min, 50 ºC for 1 min, 72 ºC for 45 s, then a final extension step at 72 ºC for 5 min. PCR positive samples were confirmed by a repeat round of PCR before sequencing to negate false positive results.

Sequencing MAdV-1 complete hexon and DNA polymerase genes from HTS data

Primers were designed (Supplementary Table S2) to span gaps between contigs obtained from MAdV-1 from HTS in the hexon and DNA polymerase gene regions. Initial long sequences were amplified using a PCR reaction mixture of 3 µL of pine marten DNA with 2 µL (5 U) LongAmp *Taq* DNA polymerase (New England Biolabs, Ipswich, Massachusetts USA), 10 µL 5x LongAmp *Taq* reaction buffer, 2 µL dNTPs (final

concentration 200 μM each dNTP), 2 μL forward primer (final concentration 200 nM), 2 μL reverse primer (final concentration 200 nM) and 29 μL H_2O.

Additional primers were then designed, and shorter sequences obtained, using a reaction mixture of 2-4 μL of DNA template, 0.2 μL (1 U) GoTaq G2 DNA polymerase (Promega), 10 μL 5x Green GoTaq reaction buffer (Promega), 1 μL dNTPs (final concentration 200 μM each dNTP), 1 μL forward primer (final concentration 200 nM), 1 μL reverse primer (final concentration 200 nM) and H_2O to a final volume of 50 μL.

The PCR reaction conditions varied for each primer set (Supplementary Table S3). Amplicons were sequenced by direct Sanger sequencing when possible (Edinburgh Genomics, Edinburgh, UK); otherwise, sequencing of cloned DNA was performed, using the pGEM-T Easy Vector System (Promega), where indicated.

Supplementary Table S1.

Summary of the mustelid samples used in the study, including the source (location) of the specimen and the adenovirus test result for samples as determined by PCR and sequencing.

ID (Species, individual ID)*	Location (Scotland, UK)	Samples positive†	Samples negative†	Samples not available‡
Pine marten 01	Portlethen, Aberdeenshire	-	Liver, kidney, lung, faeces	-
Pine marten 02	Black Isle, Ross and Cromarty	-	Liver, kidney, lung, faeces	-
Pine marten 03	Not recorded	-	Liver, kidney, lung, faeces	-
Pine marten 04	Helmsdale, Highland	-	Liver, kidney, lung, faeces	-
Pine marten 05	Not recorded	Faeces	Liver, kidney, lung	-
Pine marten 06	Not recorded	-	Liver, kidney, lung, faeces	-
Pine marten 07	Achvaich, Highland	-	Liver, kidney, lung, faeces	-
Pine marten 08	Dornoch, Highland	-	Liver, kidney, lung, faeces	-
Pine marten 09	Tarlogie, Highland	Faeces	Liver, kidney, lung	-
Pine marten 10	Not recorded	Liver, kidney, lung, faeces	-	-
Pine marten 11	Not recorded	-	Liver, kidney, lung, faeces	-
Pine marten 12	Not recorded	-	Liver, kidney, lung	Faeces
Pine marten 13	Not recorded	Liver, kidney, faeces	Lung	-
Pine marten 14	Not recorded	-	Liver, lung	Kidney, faeces
Otter 01	Shetland	-	Liver, kidney	-
Otter 02	Shetland	Kidney	Liver	-
Otter 03	Shetland	Kidney	Liver	-
Otter 04	Shetland	Liver, kidney	-	-
Otter 05	Shetland	Liver	Kidney	-
Otter 06	Shetland	Liver	Kidney	-
Otter 07	Shetland	Liver, kidney	-	-
Otter 08	Shetland	Liver, kidney	-	-
Otter 09	Shetland	Liver	Kidney	-

* Pine marten (*Martes martes*), Eurasian otter (*Lutra lutra*)
† Pine marten PCR result for MAdV-1; Eurasian otter PCR result for LAdV-1
‡ Samples routinely screened in pine martens: liver, kidney, lung, faeces; Otters: liver, kidney

Supplementary Table S2.

Summary of the primers used to sequence the MAdV-1 DNA polymerase and hexon genes.

Primer name	Sense/Antisense	Sequence (5'-3')	Target
k87_5383_pol_F	Sense	TCTACCGTGAGGGAAAGGTC	DNA Polymerase
k87_5383_pol_R	Antisense	GGTTAGTGTGGACAAACGAGA	DNA Polymerase
k87_4084_5009_F	Sense	CCCCACTGGTGAGCTGTAGA	DNA Polymerase
k87_4084_5009_R	Antisense	ATCGGGAGTTTAGGCGCTAT	DNA Polymerase
PM_pol1_AS	Antisense	ACAAGGAGTGTGGGCATGTT	DNA Polymerase
PM_pol1_S	Sense	AACATGCCCACACTCCTTGT	DNA Polymerase
PM_pol2_AS	Antisense	GATATTGCATCCTCGGATCG	DNA Polymerase
PM_pol2_S	Sense	CGATCCGAGGATGCAATATC	DNA Polymerase
PM_pol3_AS	Antisense	AGCAACTCGTTGTATGGTGCT	DNA Polymerase
PM_pol4_S	Sense	CAGCGAACCTCTTGACTAAGTTT	DNA Polymerase
PM_pol4_AS	Antisense	AAACTTAGTCAAGAGGTTCGCTG	DNA Polymerase
PM_pol5_S	Sense	CTTGACCCTGAAGCTAATGTCA	DNA Polymerase
PM_pol5_AS	Antisense	TGACATTAGCTTCAGGGTCAAG	DNA Polymerase
PM_pol6_AS	Antisense	GATGCCACTCCTGATGCTCT	DNA Polymerase
PM_pol7_S	Sense	GCCCTTGAAGGTGTAGATGC	DNA Polymerase
k87_5634_4643_F	Sense	AGCTGCAGGCGCTTATACA	Hexon
k87_5634_4643_R	Antisense	GGTCTCACGTAGGCACCGTA	Hexon
k87_3902_961_F	Sense	CTTTATGGGCGCCAAGTC	Hexon
k87_3902_961_R	Antisense	CATGACACCCTTCAAGCTGAG	Hexon
k87_5634_4643_F	Sense	AGCTGCAGGCGCTTATACA	Hexon
PM_hex1_F	Sense	AATGGCACCAAGTTCCTCTC	Hexon
PM_hex1_R	Antisense	AACTGGGAGCGGTATTTCAA	Hexon
PM_hex1_R_S	Sense	TTGAAATACCGCTCCCAGTT	Hexon
PM_hex1_R_outer	Antisense	ACGCTGTACAGAGCGGACTT	Hexon
PM_hex2_AS	Antisense	GGGCTTGTACATGTCCTCGT	Hexon
PM_hex3_S	Sense	GTGCCGTTCAGCTCAGACTT	Hexon
PM_hex4_S	Sense	GCAACCCCATCATAGACATCT	Hexon
PM_hex5_F	Sense	AAAGTTCCTCTGTGACAACTACCTC	Hexon
PM_hex5_R	Antisense	TGTTGTCGAAGACACCCAGA	Hexon
PM_hex6_AS	Antisense	TAGTTGGTACGGGTCAGGCT	Hexon
PM_hex7_AS	Antisense	GTAGTGGTGCCTGCTCATCA	Hexon
PM_hex8_F	Sense	AGCCCAGATCCAATTCACTG	Hexon
PM_hex8_R	Antisense	TGACTTTGAGCCGTGTGAAG	Hexon
PM_hex9_F	Sense	GAGGAATTTTGACCCCATGA	Hexon
PM_hex9_R	Antisense	TGGTGTACATGGGGTTCTGC	Hexon

Supplementary Table S3.

PCR reaction conditions for primer sets used to obtain sequence from MAdV-1 DNA polymerase and hexon genes.

Forward primer	Reverse primer	*Taq* polymerase	Reaction conditions *
k87_5383_pol_F	k87_5383_pol_R	LongAmp	94 ºC for 30 s, 35 x (94 for 30 s, 57 ºC for 30 s, 65 ºC for 1 min 40 s), 65 ºC for 10 min
k87_4084_5009_F	k87_4084_5009_R	LongAmp	94 ºC for 30s, 35 x (94 ºC for 30 s, 57 ºC for 30 s, 65 ºC for 1 min 40 s), 65 for 10 min
k87_5634_4643_F	k87_5634_4643_R	LongAmp	94 ºC for 30 s, 35 x (94 ºC for 30s, 57 ºC for 30s, 65 ºC for 1 min 40 s), 65 ºC for 10 min
k87_3902_961_F	k87_3902_961_R	LongAmp	94 ºC for 30 s, 35 x (94 ºC for 30 s, 57 ºC for 30 s, 65 ºC for 2 min), 65 ºC for 10 min
k87_5634_4643_F	k87_3902_961_R	LongAmp	94 ºC for 30 s, 35 x (94 ºC for 30 s, 57 ºC for 30 s, 65 ºC for 4 min), 65 ºC for 10 min
k87_5383_pol_F	PM_pol1_AS	GoTaq	95 ºC for 2 min, 35 x (95 ºC for 30 s, 52 ºC for 30 s, 72 ºC for 1 min), 72 ºC for 5 min
PM_pol1_S	PM_pol2_AS	GoTaq	95 ºC for 2 min, 35 x (95 ºC for 30 s, 52 ºC for 30 s, 72 ºC for 1 min), 72 ºC for 5 min
PM_pol2_S	PM_pol3_AS	GoTaq	95 ºC for 2 min, 35 x (95 ºC for 30 s, 52 ºC for 30 s, 72 ºC for 1 min), 72 ºC for 5 min
k87_4084_5009_F	PM_pol2_AS	GoTaq	95 ºC for 2 min, 35 x (95 ºC for 30 s, 52 ºC for 30 s, 72 ºC for 1 min, 72 ºC for 5 min
PM_pol4_S	PM_pol5_AS	GoTaq	95 ºC for 2 min, 35 x (95 ºC for 30 s, 52 ºC for 30 s, 72 ºC for 1 min 30 s), 72 ºC for 5 min
PM_pol5_S	k87_4084_5009_R	GoTaq	95 ºC for 2 min, 35 x (95 ºC for 30 s, 52 ºC for 30 s, 72 ºC for 1 min), 72 ºC for 5 min
PM_hex1_F	PM_hex1_R	GoTaq	95 ºC for 2 min, 35 x (95 ºC for 30 s, 52 ºC for 30 s, 72 ºC for 1 min), 72 ºC for 5 min
PM_hex1_R_S	PM_hex1_R_outer	GoTaq	95 ºC for 2 min, 35 x (95 ºC for 30 s, 52 ºC for 30 s, 72 ºC for 1 min), 72 ºC for 5 min
k87_3902_961_F	PM_hex2_AS	GoTaq	95 ºC for 2 min, 35 x (95 ºC for 30 s, 52 ºC for 30s, 72 ºC for 1 min 30 s), 72 ºC for 5 min
PM_hex3_S	PM_hex2_AS	GoTaq	95 ºC for 2 min, 35 x (95 ºC for 30 s, 52 ºC for 30 s, 72 ºC for 1 min), 72 ºC for 5 min
PM_hex4_S	k87_3902_961_R	GoTaq	95 ºC for 2 min, 35 x (95 ºC for 30 s, 52 ºC for 30 s, 72 ºC for 1 min), 72 ºC for 5 min
k87_5383_pol_F	PM_pol2_AS	GoTaq	95 ºC for 2 min, 35 x (95 ºC for 30 s, 52 ºC for 30 s, 72 ºC for 1 min), 72 ºC for 5 min
PM_pol1_S	PM_pol6_AS	GoTaq	95 ºC for 2 min, 35 x (95 ºC for 30 s, 52 ºC for 30 s, 72 ºC for 1 min), 72 ºC for 5 min
PM_pol7_S	PM_pol5_AS	GoTaq	95 ºC for 2 min, 35 x (95 ºC for 30 s, 52 ºC for 30 s, 72 ºC for 1 min), 72 ºC for 5 min
PM_hex5_F	PM_hex5_R	GoTaq	95 ºC for 2 min, 35 x (95 ºC for 30 s, 57 ºC for 30 s, 72 ºC for 45 s), 72 ºC for 5 min
PM_hex1_F	PM_hex7_AS	GoTaq	95 ºC for 2 min, 35 x (95 ºC for 30 s, 52 ºC for 30 s, 72 ºC for 1 min), 72 ºC for 5 min
k87_5634_4643_F	PM_hex6_AS	GoTaq	95 ºC for 2 min, 40 x (95 ºC for 30 s, 54 ºC for 30 s, 72 ºC for 1 min 40 s), 72 ºC for 5 min *
PM_hex8_F	PM_hex8_R	GoTaq	95 ºC for 2 min, 40 x (95 ºC for 30 s, 54 ºC for 30 s, 72 ºC for 1 m 40 s), 72 ºC for 5 min
PM_hex9_F	PM_hex9_R	GoTaq	95 ºC for 2 min, 40 x (95 ºC for 30 s, 52 ºC for 30 s, 72 ºC for 1 min), 72 ºC for 5 min

* Product cloned before sequencing
